# Predictive Value of Preoperative Prognostic Nutritional Index and Modified Surgical Apgar Score for Severe Postoperative Complications in Malignant Obstructive Jaundice

**DOI:** 10.3390/curroncol33090560

**Published:** 2026-09-15

**Authors:** Shiwei Wu, Jiawei Jin, Dongxing Zhang, Daobin Wang, Jiayue Zou, Xiaoyuan Hu, Jun He, Xiaofeng Xue, Lei Qin

**Affiliations:** 1 Pancreatic Disease Center, The First Affiliated Hospital of Soochow University, Suzhou 215006, China; 2 Department of General Surgery, The First Affiliated Hospital of Soochow University, Suzhou 215006, China

**Keywords:** malignant obstructive jaundice, prognostic nutritional index, modified surgical Apgar score, postoperative complications, nomogram

## Abstract

Objective: This study aimed to investigate the predictive value of preoperative prognostic nutritional index (PNI) and intraoperative modified surgical Apgar score (mSAS) for severe complications (Clavien–Dindo grade ≥ III) after curative-intent surgery in patients with malignant obstructive jaundice (MOJ) and to construct a nomogram prediction model. Methods: The clinical data of 308 patients who underwent curative-intent surgery for MOJ at the First Affiliated Hospital of Soochow University from January 2019 to January 2024 were retrospectively collected. Independent predictive factors were screened using univariate and multivariate logistic regression; the dose–response relationship between PNI and complication risk was analyzed using restricted cubic spline (RCS); subgroup analysis was conducted to evaluate the consistency of predictive effects; procedure-specific sensitivity analyses were performed separately for patients undergoing pancreaticoduodenectomy (PD) and hepatectomy with extrahepatic bile duct resection (HCAE); a nomogram was constructed based on the independent predictive factors, and the performance of the model was evaluated using receiver operating characteristic (ROC), calibration curve, and decision curve analysis (DCA). Results: Among the 308 patients, 65 (21.1%) had severe complications. The PNI (40.30 ± 4.19 vs. 45.91 ± 4.75) and mSAS (5.28 ± 1.28 vs. 7.28 ± 1.34) in the severe complication group were significantly lower than those in the non-severe complication group (all *p* < 0.001). After multivariable adjustment including surgical approach, higher PNI (OR = 0.73, 95% CI 0.66–0.82, *p* < 0.001) and higher mSAS (OR = 0.30, 95% CI 0.21–0.44, *p* < 0.001) were independently associated with lower odds of severe complications. The quartile analysis of PNI showed a graded inverse association (*p* for trend < 0.001). Multivariable-adjusted RCS analysis demonstrated a significant overall inverse association between PNI and severe complication risk (*p* for overall < 0.001), with no significant evidence of nonlinearity (*p* for nonlinearity = 0.425). In procedure-specific sensitivity analyses, the PNI–mSAS model achieved an AUC of 0.959 (95% CI 0.921–0.997) in the PD subgroup and 0.901 (95% CI 0.837–0.965) in the HCAE subgroup. The AUC of the nomogram prediction model was 0.924; the calibration curve indicated good agreement, and DCA suggested potential clinical utility. Conclusion: Preoperative PNI and intraoperative mSAS are independently associated with severe complications after curative-intent surgery in patients with MOJ. The PNI–mSAS nomogram showed good internal discrimination and calibration and may assist perioperative risk stratification; however, external validation is required before routine clinical application.

## Simple Summary

Malignant obstructive jaundice is associated with substantial postoperative risk after major pancreatic and hepatobiliary surgery. This study evaluated whether two simple perioperative measures—the preoperative prognostic nutritional index (PNI), reflecting nutritional and immune reserve, and the intraoperative modified Surgical Apgar Score (mSAS), reflecting physiological stress—could help identify patients at higher risk of severe postoperative complications. Among 308 patients, lower PNI and lower mSAS were independently associated with Clavien–Dindo grade ≥ III complications. A model combining PNI and mSAS showed good internal discrimination, and procedure-specific analyses demonstrated high discriminatory performance in both pancreaticoduodenectomy and hepatectomy with extrahepatic bile duct resection groups. These findings suggest that integrating preoperative biological reserve with intraoperative physiological information may support perioperative risk stratification.

## 1. Introduction

Malignant obstructive jaundice (MOJ) is a clinical syndrome caused by malignant tumors of the biliary system (such as cholangiocarcinoma, pancreatic head cancer, gallbladder cancer, ampullary peri-carcinoma, etc.) that compress or invade the bile ducts. It is characterized by progressively worsening obstructive jaundice. Due to the obstruction of bile excretion, patients often suffer from liver function impairment, intestinal barrier dysfunction, endotoxemia, and immune function suppression [1,2,3,4]. Pancreaticoduodenectomy (PD) and hepatectomy with extrahepatic bile duct resection (HCAE), among others, are currently the only potentially curative treatments for MOJ. However, such surgeries are traumatic and complex [5], and this group often has multiple risk factors such as hyperbilirubinemia, decreased liver reserve function, and malnutrition. The incidence of severe complications (Clavien–Dindo grade ≥ III) after surgery is as high as 15–30% [6,7], which is significantly higher than that in the general abdominal surgery population. Severe complications not only prolong hospital stays and increase economic burdens but also may delay adjuvant treatment and affect long-term prognosis [8]. Therefore, accurately identifying high-risk patients with severe complications before and during surgery is important for intraoperative management and perioperative monitoring.

The prognostic nutritional index (PNI) was proposed by Onodera et al. [9] in 1984 as a composite indicator calculated from serum albumin and peripheral blood lymphocyte counts. This index comprehensively reflects the body’s nutritional reserve and immune status and has been widely used in the prediction of postoperative complications and prognosis in various digestive system tumors [10,11,12]. In patients with obstructive jaundice, due to bile stasis, liver protein synthesis function is impaired, and intestinal flora translocation leads to immune activation and consumption, potentially making PNI more sensitive in reflecting the overall reserve function of patients compared to the general surgical population. In the field of biliary tumors, Wang et al. [13] found that PNI is an independent predictor of overall survival in patients with resectable biliary cancer, but its predictive value is affected by the presence of obstructive jaundice. However, the predictive value of PNI for severe complications after curative-intent surgery for MOJ and the dose–response relationship between PNI and complication risk have not been systematically and deeply evaluated.

On the other hand, intraoperative assessment is also important. The Surgical Apgar Score (SAS) was proposed by Gawande et al. [14] in 2007 and is scored based on three indicators: estimated blood loss during surgery, minimum heart rate, and minimum mean arterial pressure. Miki et al. [15] proposed the Modified Surgical Apgar Score (mSAS) in 2014, with threshold values adjusted according to surgical type, and its predictive value has been preliminarily verified in liver, gallbladder, and pancreas surgeries, gastrointestinal surgeries, etc. [16,17,18]. The mSAS is simple and can be calculated immediately after surgery. However, evidence for the use of mSAS in patients with obstructive jaundice remains limited.

Based on the above background, this study aims to (1) evaluate whether preoperative PNI and mSAS are independent predictors of severe complications (Clavien–Dindo grade ≥ III) after curative-intent surgery for MOJ; (2) use restricted cubic spline (RCS) analysis to characterize the dose–response relationship between PNI and complication risk; (3) evaluate the consistency of the predictive effect of PNI in different clinical subgroups through subgroup analysis; and (4) construct and validate a nomogram prediction model integrating PNI and mSAS to provide a visual tool for perioperative risk stratification.

## 2. Materials and Methods

### 2.1. Research Design and Subjects

The clinical data of patients who underwent curative-intent surgery for MOJ in the Department of General Surgery, The First Affiliated Hospital of Soochow University, from January 2019 to January 2024 were retrospectively collected (*n* = 308). The enrolled patients were screened according to the prespecified inclusion and exclusion criteria. The research protocol was approved by the ethics committee of our hospital, and the ethics approval number was 2026846. The patients were divided into the occurrence group and the non-occurrence group based on whether they developed Clavien–Dindo grade ≥ III complications within 90 days after surgery.

Inclusion criteria: (1) meeting the diagnostic criteria for obstructive jaundice; (2) not receiving neoadjuvant therapy and undergoing curative-intent surgery; (3) pathologically diagnosed as malignant tumors; and (4) complete data and information. Exclusion criteria: (1) obstructive jaundice caused by benign diseases; (2) already having distant metastasis or combined with other malignant tumors; (3) not receiving surgical treatment or only receiving palliative surgery; and (4) missing or incomplete clinical data.

### 2.2. Outcome Variables

The main outcome measure of this study was severe postoperative complications, which were determined strictly following the Clavien–Dindo classification system [6] (Table 1). The specific types of complications included intra-abdominal infection, biliary fistula, pancreatic fistula, intra-abdominal hemorrhage, incision infection, and liver failure. The final diagnosis of all complications was based on the patient’s clinical symptoms and signs, combined with laboratory tests, imaging examination results, and confirmation through secondary surgical exploration when necessary.

For the present predictive analyses, the primary endpoint was overall major morbidity defined as Clavien–Dindo grade ≥ III within 90 days; procedure-specific complication classification systems were not used as separate model outcomes.

### 2.3. Key Variables

PNI was calculated from peripheral blood lymphocyte count (×10^9^/L) and serum albumin level (g/L). The calculation formula for PNI is as follows:

$$\mathrm{PNI}=Albumin\;level\left(g/L\right)+5\times \mathrm{Lymphocyte}(\times 10^{9}/L)$$

Laboratory values from the blood test closest to surgery and obtained within 7 days before surgery were used to calculate PNI. For patients who underwent preoperative biliary drainage, post-drainage laboratory measurements were used. In patients with active cholangitis or other clinically evident inflammatory or infectious conditions, PNI was calculated using the most recent laboratory measurements obtained after clinical control of the infection and before surgery.

The modified Surgical Apgar Score (mSAS) was calculated using three intraoperative variables: the lowest heart rate, the lowest mean arterial pressure, and estimated blood loss. For the lowest heart rate, >85, 76–85, 66–75, 56–65, and ≤55 beats/min were assigned 0, 1, 2, 3, and 4 points, respectively. For the lowest mean arterial pressure, <40, 40–54, 55–69, and ≥70 mmHg were assigned 0, 1, 2, and 3 points, respectively. For estimated blood loss, >1000 mL or intraoperative transfusion, 601–1000 mL, 101–600 mL, and ≤100 mL were assigned 0, 1, 2, and 3 points, respectively. The total mSAS therefore ranged from 0 to 10 points, with a higher score indicating a more favorable intraoperative physiological profile [15].

### 2.4. Covariates

The collected covariates included age, sex, body mass index (BMI), hypertension, diabetes, cardiovascular disease, preoperative biliary drainage method (none, PTBD, and EBD), presence or absence of high biliary obstruction, surgical approach (PD and HCAE), laparoscopic surgery (Yes or No), and preoperative total bilirubin (TBIL).

### 2.5. Statistical Methods

Normally distributed continuous variables were expressed as mean ± standard deviation (SD), whereas skewed variables were expressed as median and interquartile range (IQR). Between-group comparisons were performed using the *t*-test or analysis of variance for normally distributed data and the rank-sum test for skewed data. Categorical variables were expressed as *n* (%) and compared using the chi-square test or Fisher’s exact test. Univariate and multivariate logistic regression analyses were performed, with results expressed as odds ratios (ORs) and 95% confidence intervals (CIs). Variables included in the multivariate model were sex, age, preoperative biliary drainage method (none, PTBD, and EBD), mSAS, PNI, TBIL, and surgical approach (PD vs. HCAE).

Restricted cubic spline (RCS) regression was used to model the dose–response relationship between PNI and the risk of severe complications using three knots (the 10th, 50th, and 90th percentiles). The overall association and nonlinearity were tested [19]. The RCS model was adjusted for the same covariates as the primary multivariable logistic regression model (sex, age, preoperative biliary drainage method, mSAS, TBIL, and surgical approach), with PNI entered as spline terms. Subgroup analyses were conducted stratified by age (<65 years vs. ≥65 years), sex, BMI (<24 kg/m^2^ vs. ≥24 kg/m^2^), hypertension, diabetes, cardiovascular disease, preoperative biliary drainage method (none, PTBD, and EBD), surgical approach (PD and HCAE), and laparoscopic surgery (Yes and No).

Because mSAS is derived from intraoperative variables, the combined PNI–mSAS model is intended for perioperative or early postoperative risk stratification rather than as a purely preoperative prediction tool.

To assess the robustness of the primary findings to heterogeneity in surgical procedures, a procedure-specific sensitivity analysis was performed. Patients undergoing PD and HCAE were analyzed separately, and logistic regression models incorporating PNI and mSAS were fitted within each surgical subgroup. Model discrimination was evaluated using the area under the ROC curve (AUC) with 95% confidence intervals.

A nomogram prediction model was constructed using PNI and mSAS. Model performance was evaluated in terms of (1) discrimination, using the area under the receiver operating characteristic (ROC) curve (AUC); (2) calibration, using a calibration curve generated with bootstrap resampling (1000 resamples) and the Hosmer–Lemeshow goodness-of-fit test (*p* > 0.05 indicating good calibration); and (3) clinical utility, using decision curve analysis (DCA) to calculate net benefit across different threshold probabilities. All statistical analyses were performed using R version 4.4.1 with two-sided tests and a significance level of α = 0.05. During the preparation of this manuscript, the author(s) used ChatGPT (OpenAI, GPT-4) for the purposes of language editing and improving readability. The authors have reviewed and edited the output and take full responsibility for the content of this publication.

## 3. Results

### 3.1. Baseline Characteristics

A total of 308 patients who underwent curative-intent surgery for MOJ were included. Among them, 65 patients (21.1%) experienced severe postoperative complications (Clavien–Dindo grade ≥ III), and 243 cases (78.9%) did not. There were no statistically significant differences between the two groups in terms of age (65.76 ± 8.47 vs. 66.46 ± 8.99 years, *p* = 0.559), BMI (22.63 ± 3.02 vs. 22.72 ± 3.34 kg/m^2^, *p* = 0.836), hypertension (39.9% vs. 43.1%, *p* = 0.750), diabetes (17.3% vs. 16.9%, *p* = 1.000), and cardiovascular diseases (11.1% vs. 15.4%, *p* = 0.467). There was a significant between-group difference in the distribution of preoperative biliary drainage (*p* = 0.014), and the proportion of EBD in the severe complication group was significantly higher than that in the non-severe complication group (13.8% vs. 4.1%). The preoperative PNI (40.30 ± 4.19 vs. 45.91 ± 4.75, *p* < 0.001) and intraoperative mSAS (5.28 ± 1.28 vs. 7.28 ± 1.34, *p* < 0.001) in the severe complication group were significantly lower than those in the non-severe complication group. There was no statistically significant difference in TBIL between the two groups (*p* = 0.255). When stratified by principal surgical category, severe complications occurred in 40 of 210 patients undergoing PD (19.0%) and in 25 of 98 patients undergoing HCAE (25.5%) (Table 2).

### 3.2. Multivariate Logistic Regression Analysis

#### 3.2.1. PNI as a Continuous Variable

In the univariate analysis, PNI (OR = 0.72, 95% CI 0.66–0.79, *p* < 0.001) and mSAS (OR = 0.32, 95% CI 0.23–0.43, *p* < 0.001) were significantly associated with severe postoperative complications. EBD (vs. no drainage) was also associated with severe complications (OR = 3.92, 95% CI 1.46–10.55, *p* = 0.007). After additional adjustment for surgical approach, PNI (OR = 0.73, 95% CI 0.66–0.82, *p* < 0.001) and mSAS (OR = 0.30, 95% CI 0.21–0.44, *p* < 0.001) remained independently associated with lower odds of severe complications. Surgical approach (PD vs. HCAE) was not independently associated with severe complications (OR = 0.87, 95% CI 0.36–2.13, *p* = 0.761). Specifically, each 1-unit increase in PNI was associated with approximately 27% lower odds of severe complications, and each 1-point increase in mSAS was associated with approximately 70% lower odds (Table 3).

#### 3.2.2. PNI Quartile Group Analysis

To further investigate the relationship between PNI and the risk of severe complications, patients were divided into quartiles based on PNI levels, with Q1 as the reference group. After adjusting for covariates, including surgical approach, the risks in Q2 (OR = 0.42, 95% CI 0.17–0.99, *p* = 0.049), Q3 (OR = 0.14, 95% CI 0.05–0.42, *p* < 0.001), and Q4 (OR = 0.03, 95% CI 0.01–0.15, *p* < 0.001) showed a graded inverse association, with reductions observed across Q2–Q4 (*p* for trend < 0.001) (Table 4).

### 3.3. Dose–Response Relationship Analysis

**Figure 1:**
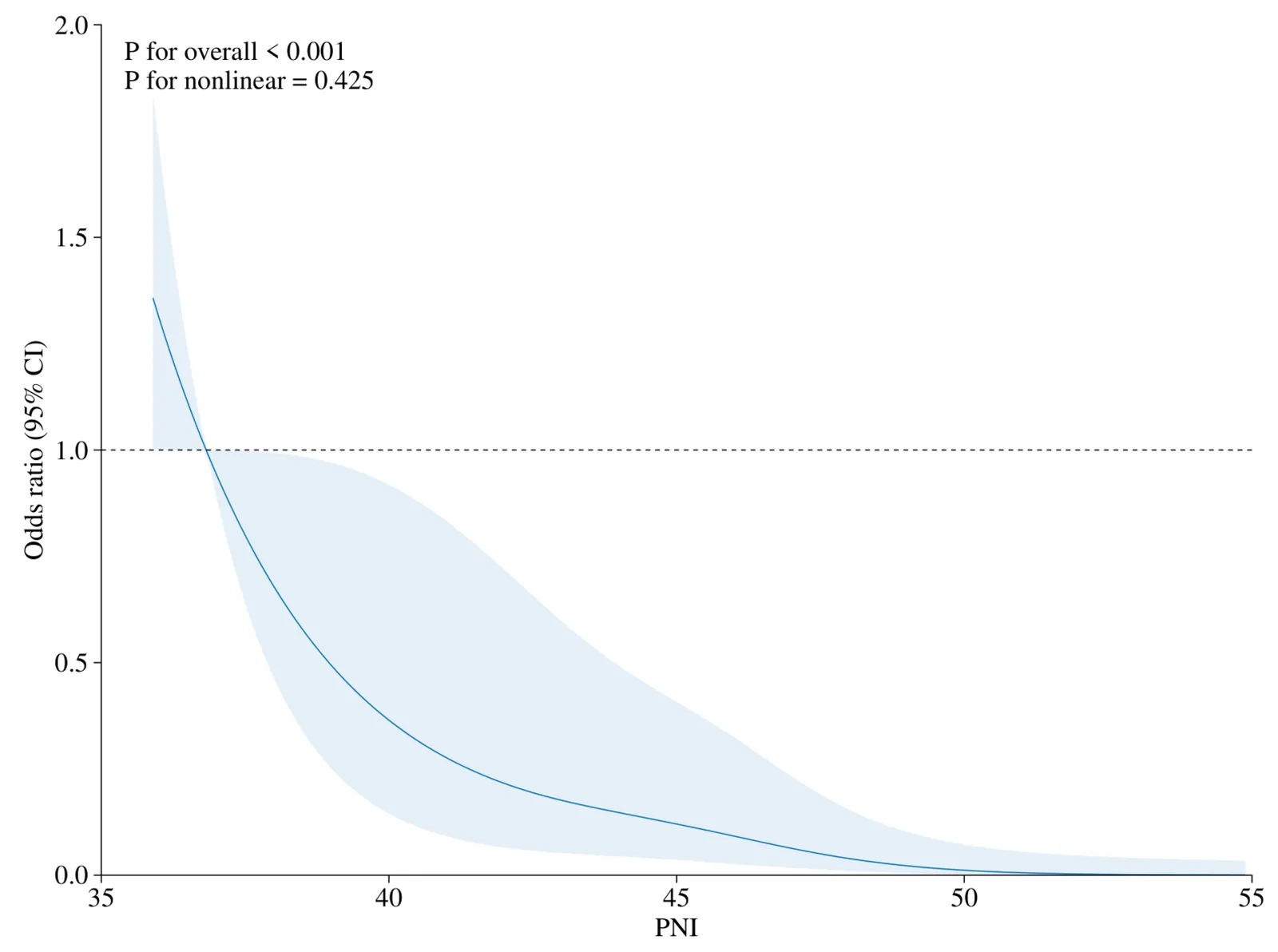
Multivariable-adjusted restricted cubic spline analysis of the association between PNI and the risk of severe postoperative complications. The overall association was statistically significant (*p* for overall < 0.001), whereas no significant nonlinearity was detected (*p* for nonlinearity = 0.425). Shading and dashed lines represent the 95% confidence interval.

Multivariable-adjusted RCS analysis demonstrated a significant overall inverse association between PNI and the risk of severe postoperative complications (*p* for overall < 0.001). However, there was no statistically significant evidence of nonlinearity (*p* for nonlinearity = 0.425). The estimated risk of severe complications decreased progressively with increasing PNI levels, supporting an inverse association between preoperative PNI and postoperative complication risk (Figure 1).

### 3.4. Subgroup Analysis

**Figure 2:**
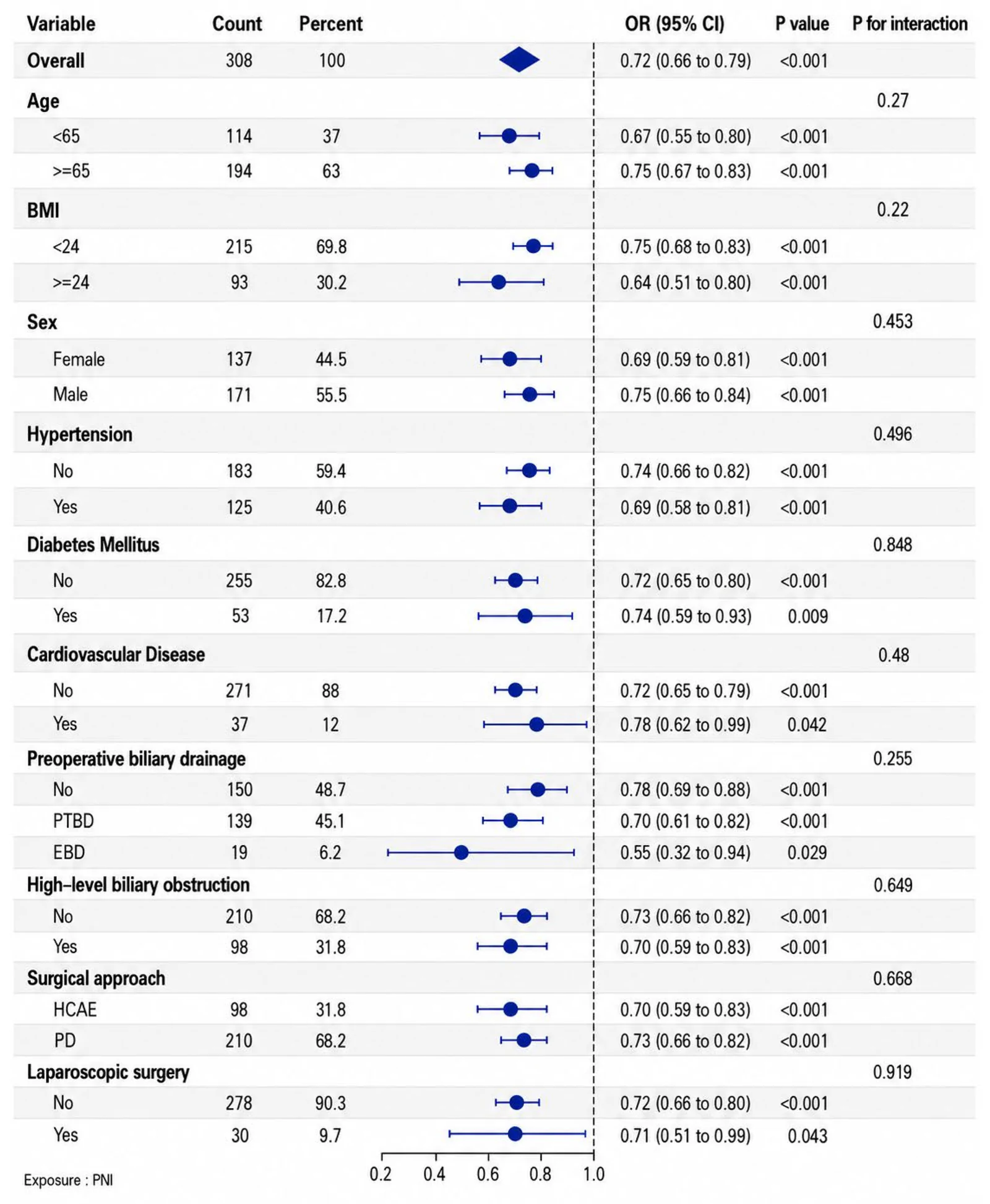
Forest plot of subgroup analysis for the association between PNI and severe complications.

To further evaluate the predictive stability of PNI across different populations, we conducted subgroup analyses based on age, BMI, sex, comorbidities, and surgical factors. The results (Figure 2) showed an inverse association between PNI and severe postoperative complications across all examined subgroups (all odds ratios < 1, with *p* values < 0.05). Specifically, the association was similar between patients aged <65 years (OR = 0.67, 95% CI 0.55–0.80) and those aged ≥65 years (OR = 0.75, 95% CI 0.67–0.83); likewise, patients with BMI < 24 kg/m^2^ (OR = 0.75, 95% CI 0.68–0.83) and those with BMI ≥ 24 kg/m^2^ (OR = 0.64, 95% CI 0.51–0.80) exhibited comparable trends. The inverse association remained directionally consistent regardless of sex, hypertension, diabetes, cardiovascular disease, surgical approach, preoperative biliary drainage, or laparoscopic surgery. None of the interaction *p* values reached statistical significance, indicating no statistically significant effect modification across the examined subgroups.

### 3.5. Procedure-Specific Sensitivity Analysis

To assess the robustness of the PNI–mSAS model across different surgical procedures, separate logistic regression models were constructed for patients undergoing PD and HCAE. In the PD subgroup, PNI (OR = 0.75, 95% CI 0.64–0.88, *p* < 0.001) and mSAS (OR = 0.09, 95% CI 0.04–0.21, *p* < 0.001) were significantly associated with severe postoperative complications, and the combined model achieved an AUC of 0.959 (95% CI 0.921–0.997). In the HCAE subgroup, PNI (OR = 0.74, 95% CI 0.63–0.87, *p* < 0.001) and mSAS (OR = 0.54, 95% CI 0.37–0.79, *p* = 0.001) also remained significantly associated with severe complications, with an AUC of 0.901 (95% CI 0.837–0.965). These findings indicate that the predictive performance of the PNI–mSAS model remained high when the two surgical procedures were analyzed separately (Supplementary Table S2).

### 3.6. Construction and Evaluation of the Nomogram Prediction Model

**Figure 3:**
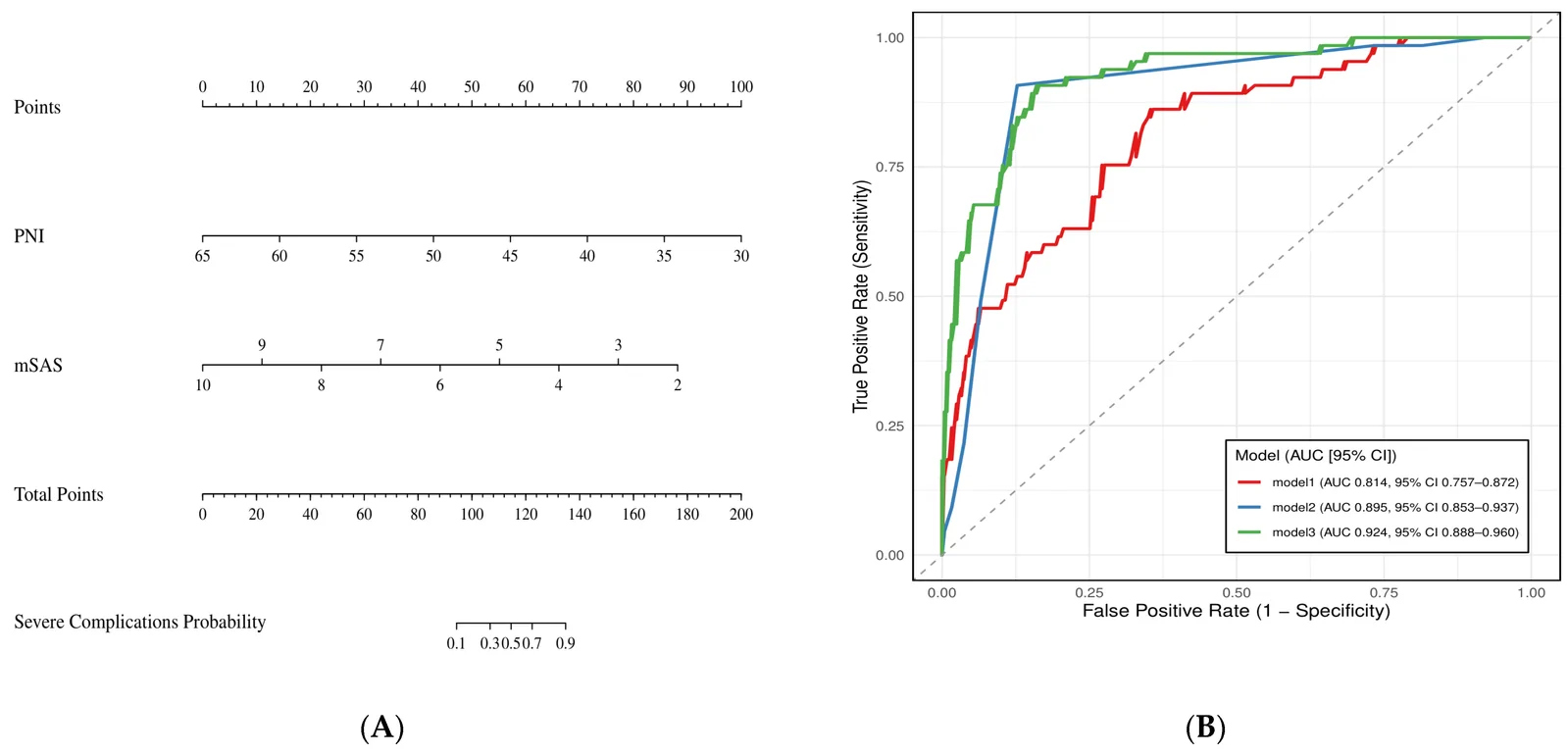

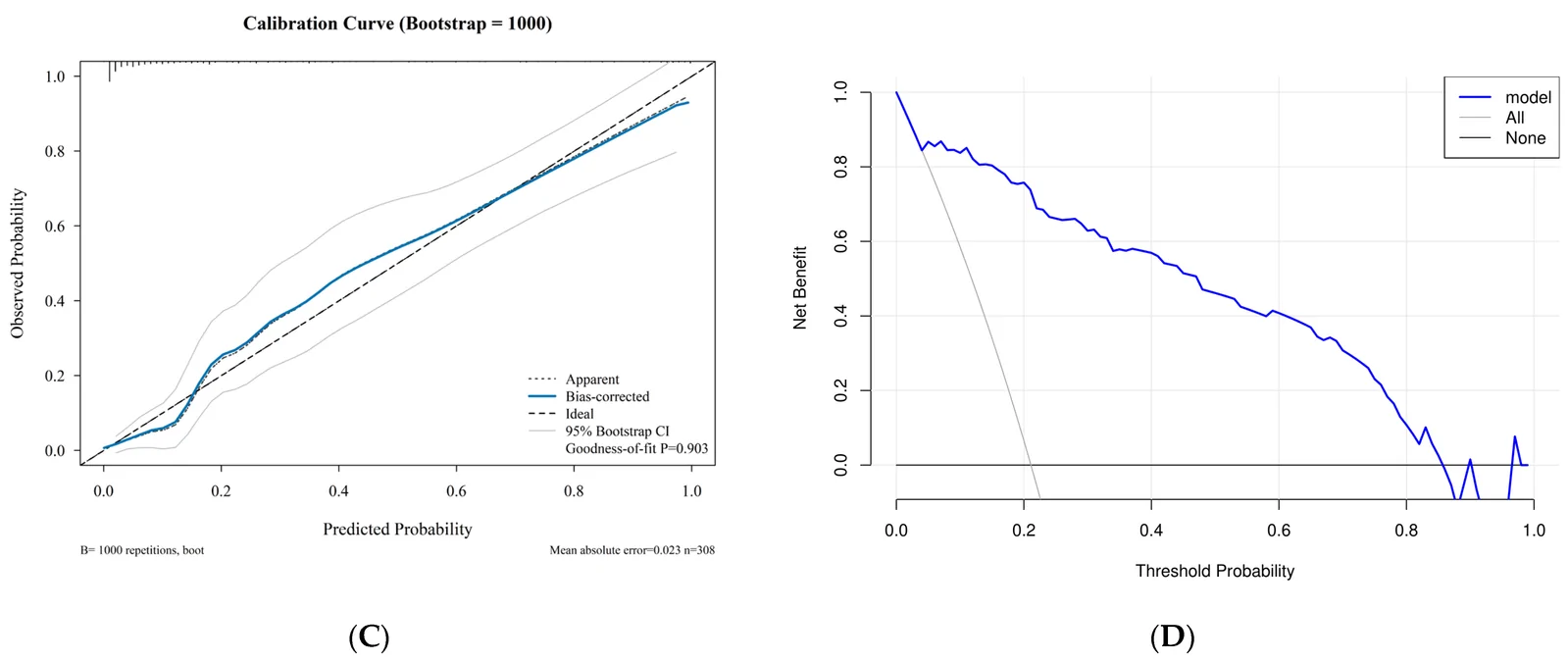
Construction and validation of a nomogram for predicting severe complications after MOJ: (**A**) Nomogram for predicting the probability of severe complications after MOJ. (**B**) Receiver operating characteristic (ROC) curves of PNI, mSAS, and the combined prediction model (PNI + mSAS) for predicting severe complications after MOJ. (**C**) Calibration curve of the nomogram-predicted probability versus actual frequency. (**D**) Decision curve analysis (DCA) of the nomogram.

A nomogram prediction model was developed using PNI and mSAS. Each predictor was assigned a score based on its regression coefficient, and the total score was obtained by summing individual scores and corresponded to the predicted probability of severe complications. Regarding discrimination, ROC curve analysis showed that the combined model (PNI + mSAS) had an AUC of 0.924, significantly outperforming either PNI or mSAS alone (DeLong test, all *p* < 0.05), indicating excellent discriminatory ability. In terms of calibration, the calibration curve demonstrated good agreement between predicted probabilities from the nomogram and observed probabilities, with a Hosmer–Lemeshow test *p* > 0.05, suggesting strong calibration performance. For clinical utility, decision curve analysis (DCA) showed a positive net benefit across threshold probabilities of approximately 5% to 80%, suggesting potential clinical utility within this range (Figure 3).

## 4. Discussion

This study systematically evaluated the associations of preoperative PNI and intraoperative mSAS with severe postoperative complications in 308 patients undergoing curative-intent surgery for MOJ and developed a visual nomogram prediction model. Key findings include the following: (1) both PNI and mSAS were independently associated with severe postoperative complications after curative-intent surgery for MOJ; (2) RCS analysis demonstrated a significant overall inverse association between PNI and complication risk, without statistically significant evidence of nonlinearity; (3) procedure-specific sensitivity analyses showed that the PNI–mSAS model retained high discrimination in both the PD and HCAE subgroups; and (4) the nomogram based on PNI and mSAS demonstrated good internal discrimination, calibration, and potential clinical utility.

The biological basis of PNI as a predictor warrants further exploration. This index integrates information from two dimensions—nutritional status (serum albumin) and immune function (lymphocyte count)—and serves as a composite indicator reflecting overall nutritional and immune status. Its predictive value has been confirmed across various malignancies [13,20,21]. Serum albumin is not only a classic marker of visceral protein reserves but also plays multiple physiological roles, including maintaining plasma colloid osmotic pressure, binding and transporting fatty acids and drugs, and scavenging free radicals [10]. Hypoalbuminemia reflects protein-energy malnutrition and chronic catabolism, directly affecting critical wound healing processes such as collagen synthesis, fibroblast proliferation, and neoangiogenesis [21,22,23,24]. In patients with obstructive jaundice, bile stasis leads to reduced intestinal bile content, impairing fat liquefaction and absorption of fat-soluble vitamins, thereby worsening nutritional status. Meanwhile, biliary obstruction-induced translocation of gut microbiota and endotoxemia further deplete immune resources. Thus, PNI may be particularly sensitive in reflecting overall reserve capacity in this specific patient population. Notably, the PNI level in the severe complication group (40.30) in this study was significantly higher than the cutoff value of 36.7 used by Zakosek et al. [25] to predict 60-day survival, suggesting that different study endpoints (postoperative complications vs. short-term survival) may require different PNI thresholds. Recent evidence from retroperitoneal sarcoma has also examined PNI together with skeletal muscle index in relation to postoperative and oncological outcomes, providing additional contemporary context for the role of nutritional and body-composition assessment in patients undergoing major oncologic surgery [26].

Importantly, PNI should not be interpreted as a purely nutritional marker in malignant obstructive jaundice. Because it combines serum albumin and lymphocyte count, a low PNI may also reflect systemic inflammation, cholestasis-related hepatic dysfunction, cancer-related biological burden, and reduced overall physiological reserve. Accordingly, the present findings support PNI as a prognostic marker of biological reserve rather than evidence that modifying the PNI value itself will improve postoperative outcomes.

In this study, PNI maintained a significant independent association with severe postoperative complications after adjustment for multiple clinical covariates, including surgical approach (OR = 0.73, 95% CI 0.66–0.82), indicating that the risk information captured by PNI is at least partially independent of conventional clinical and procedure-related factors. This finding aligns with results from multiple studies in gastrointestinal oncology [27,28]. Quartile analysis revealed a graded inverse association with increasing PNI levels (*p* for trend < 0.001), with the adjusted association reaching statistical significance from Q2 onward. Q4 showed markedly lower odds compared with Q1 (OR = 0.03). RCS analysis further supported an inverse association between PNI and the risk of severe postoperative complications. Although the estimated risk appeared to decline more prominently at lower PNI levels, the test for nonlinearity was not statistically significant (*p* = 0.425). Therefore, the present data do not support a specific PNI threshold or a definitive nonlinear dose–response pattern. Lower PNI may help identify patients at higher postoperative risk, but the optimal cutoff value and any role in guiding nutritional intervention require prospective validation.

The independent association of mSAS with severe postoperative complications (OR = 0.30, 95% CI 0.21–0.44, *p* < 0.001) was robustly confirmed in this study. This finding is consistent with the study by Miki et al. [15] on gastrectomy, which identified mSAS ≤ 6 as an independent risk factor for severe complications. Houqiong et al. [18] also reported the predictive value of mSAS for postoperative complications in robotic rectal cancer surgery (AUC = 0.740). Since its introduction by Gawande et al. [14], the SAS scoring system has been widely validated across multiple surgical fields [16,17,18,29]. The three components of mSAS—blood loss, lowest heart rate, and lowest mean arterial pressure—essentially reflect the integrated response of intraoperative sympathetic–adrenal system stress and circulatory compensatory capacity. In patients with obstructive jaundice, hyperbilirubinemia can impair myocardial contractility and cause abnormal vascular tone, further reducing their tolerance to hemodynamic fluctuations during surgery. Notably, the predictive effect of mSAS is independent of preoperative PNI, indicating that even with adequate preoperative nutritional preparation, unforeseen intraoperative hemodynamic changes may still significantly affect patient outcomes. This finding underscores the importance of integrating both preoperative and intraoperative risk factors.

mSAS should also not be interpreted as an exclusively patient-related physiological measure. Its components, particularly blood loss and intraoperative hemodynamic instability, may partly capture operative complexity, tumor extent, vascular involvement, technical difficulty, and procedure duration. Thus, the association between mSAS and postoperative morbidity likely reflects both physiological tolerance and characteristics of the operation itself. Importantly, PNI and mSAS remained independently associated with severe complications after the surgical approach (PD vs. HCAE) was included in the primary multivariable model, suggesting that the main associations were not explained solely by differences between the two major procedures.

The clinical advantage of combining PNI and mSAS lies in their complementary assessment of overall surgical tolerance from different temporal (preoperative vs. intraoperative) and biological dimensions (nutritional–immune reserve vs. physiological stress response). Together, they form a comprehensive risk profile spanning the preoperative to intraoperative period. ROC analysis confirmed that the combined model (AUC = 0.924) significantly outperformed either predictor alone (all *p* < 0.05 by the DeLong test). Calibration curves and DCA further supported the potential value of the “baseline risk + stress exposure” prediction framework. These findings are consistent with previous studies suggesting that nutritional status should be considered together with procedure-related and other perioperative factors for more comprehensive postoperative risk stratification [30,31]. Because PD and HCAE differ substantially in operative complexity and complication profiles, we additionally performed procedure-specific sensitivity analyses. PNI and mSAS remained significantly associated with severe postoperative complications in both surgical subgroups, while the PNI–mSAS model retained high discrimination in the PD (AUC = 0.959) and HCAE (AUC = 0.901) groups. These findings suggest that the observed predictive associations were not solely attributable to a single surgical procedure, although larger procedure-specific cohorts and external validation remain necessary.

Because mSAS becomes available only during or toward the end of the operation, the PNI–mSAS nomogram should be interpreted as a perioperative or early postoperative risk-stratification tool, with a potential role in identifying patients who may warrant closer postoperative surveillance, rather than as a purely preoperative prediction model.

The finding regarding endoscopic biliary drainage should be interpreted cautiously. EBD was associated with severe complications in univariable analysis (OR = 3.92, 95% CI 1.46–10.55, *p* = 0.007), but the association was no longer statistically significant after multivariable adjustment including surgical approach (OR = 2.16, 95% CI 0.28–16.54, *p* = 0.458). Only 19 patients underwent EBD, and the wide confidence interval indicates substantial imprecision. Confounding by indication is also plausible because patients selected for drainage may differ in obstruction severity, infection status, and overall clinical condition. Therefore, no causal conclusion regarding the drainage method can be drawn from these data.

From a clinical perspective, a low PNI should therefore be viewed as a signal for comprehensive multidisciplinary assessment rather than as an isolated laboratory target. Formal nutritional assessment, evaluation of body composition or sarcopenia when appropriate, correction of potentially reversible nutritional deficits, and individualized prehabilitation may be considered within broader perioperative optimization; whether these strategies improve outcomes specifically in malignant obstructive jaundice requires prospective evaluation.

Because the present analysis focused on overall Clavien–Dindo grade ≥ III morbidity rather than complication-specific graded endpoints, it cannot determine whether PNI or mSAS is preferentially associated with pancreatic fistula, hemorrhage, liver failure, infectious complications, or other specific events. Future procedure-specific studies should incorporate standardized complication definitions and sufficient event numbers for subtype-specific analyses.

This study has several limitations. First, the single-center retrospective design may introduce selection bias and residual confounding. Although we adjusted for multiple known confounders, unmeasured confounding cannot be entirely ruled out, and the findings require validation in multicenter prospective cohorts. Second, although the number of events was adequate for the parsimonious two-predictor nomogram, the number of outcome events was relatively limited for the fully adjusted multivariable analyses, particularly for categorical covariates and subgroup analyses. Therefore, some estimates may remain unstable and require confirmation in larger cohorts. Third, bootstrap resampling was used to assess calibration; however, model development and evaluation were performed in the same cohort. Therefore, the apparent model performance may be optimistic, and external validation is needed before routine clinical application. Fourth, the study population included different major surgical procedures with distinct operative complexity and complication profiles. Although the surgical approach was included in the primary multivariable model and procedure-specific sensitivity analyses showed high model discrimination in both PD and HCAE subgroups, residual procedure-specific heterogeneity may remain. Fifth, the optimal cutoff value for PNI may vary across populations and requires multicenter confirmation. Sixth, mSAS relies on accurate intraoperative documentation, and retrospective data may be subject to recording bias.

Preoperative PNI and intraoperative mSAS were independently associated with severe complications following curative surgery in patients with MOJ, reflecting complementary dimensions of nutritional–immune reserve and intraoperative physiological stress. The nomogram incorporating PNI and mSAS demonstrated good internal discrimination and calibration and may assist individualized perioperative risk stratification. Patients with lower preoperative PNI may represent a population at increased risk of severe postoperative complications and may warrant closer perioperative assessment. However, this retrospective observational study cannot establish that increasing PNI through nutritional or immune-modulating interventions would reduce complication risk, and no specific intervention threshold can be inferred from the present data. Prospective multicenter studies with external validation are warranted to confirm the generalizability and clinical utility of this model.

## Figures and Tables

**Table 1:** Clavien–Dindo classification of postoperative complications.

Grades	Definition
I	Any deviation from the normal postoperative course without the need for pharmacological treatment or surgical, endoscopic, and radiological interventions. Allowed therapeutic regimens include drugs such as antiemetics, antipyretics, analgesics, diuretics, and electrolytes, and physiotherapy. This grade also includes wound infections opened at the bedside.
II	Requiring pharmacological treatment with drugs other than those allowed for grade I complications. Blood transfusions and total parenteral nutrition are also included.
III	Requiring surgical, endoscopic, or radiological intervention.
IV	Life-threatening complication (including CNS complications) requiring IC/ICU management.
V	Death of a patient.

**Table 2:** Comparison of baseline characteristics between the two groups.

Characteristic		Overall (N = 308)	Non-Severe (N = 243)	Severe (N = 65)	*p*
Sex (*n*%)	Female	137 (44.5)	115 (47.3)	22 (33.8)	0.072
	Male	171 (55.5)	128 (52.7)	43 (66.2)	
Hypertension (*n*%)	No	183 (59.4)	146 (60.1)	37 (56.9)	0.75
	Yes	125 (40.6)	97 (39.9)	28 (43.1)	
Diabetes mellitus (*n*%)	No	255 (82.8)	201 (82.7)	54 (83.1)	1
	Yes	53 (17.2)	42 (17.3)	11 (16.9)	
Cardiovascular disease (*n*%)	No	271 (88.0)	216 (88.9)	55 (84.6)	0.467
	Yes	37 (12.0)	27 (11.1)	10 (15.4)	
Preoperative biliary drainage (*n*%)	No	150 (48.7)	122 (50.2)	28 (43.1)	0.014
	PTBD	139 (45.1)	111 (45.7)	28 (43.1)	
	EBD	19 (6.2)	10 (4.1)	9 (13.8)	
High-level biliary obstruction (*n*%)	No	210 (68.2)	170 (70.0)	40 (61.5)	0.252
	Yes	98 (31.8)	73 (30.0)	25 (38.5)	
Surgical approach (*n*%)	PD	210 (68.2)	170 (70.0)	40 (61.5)	0.252
	HCAE	98 (31.8)	73 (30.0)	25 (38.5)	
Laparoscopic surgery (*n*%)	No	278 (90.3)	218 (89.7)	60 (92.3)	0.695
	Yes	30 (9.7)	25 (10.3)	5 (7.7)	
Age (years, mean (SD))		65.91 (8.57)	65.76 (8.47)	66.46 (8.99)	0.559
BMI (kg/m^2^, mean (SD))		22.65 (3.09)	22.63 (3.02)	22.72 (3.34)	0.836
PNI (mean (SD))		44.72 (5.17)	45.91 (4.75)	40.30 (4.19)	<0.001
mSAS (mean (SD))		6.86 (1.56)	7.28 (1.34)	5.28 (1.28)	<0.001
TBIL (μmol/L, mean (SD))		127.13 (92.50)	124.02 (92.53)	138.75 (92.16)	0.255

**Table 3:** Univariate and multivariate logistic regression analysis of severe postoperative complications.

Characteristic	Univariable OR (95%CI)	*p*	Multivariable OR (95%CI)	*p*
PNI	0.72 (0.66~0.79)	<0.001	0.73 (0.66–0.82)	<0.001
mSAS	0.32 (0.23~0.43)	<0.001	0.30 (0.21–0.44)	<0.001
Sex (Male vs. Female)	1.76 (0.99~3.11)	0.054	2.19 (0.92–5.22)	0.077
Preoperative biliary drainage				
PTBD vs. No	1.10 (0.61~1.97)	0.751	0.66 (0.28–1.56)	0.340
EBD vs. No	3.92 (1.46~10.55)	0.007	2.16 (0.28–16.54)	0.458
Age	1.01 (0.98~1.04)	0.558	1.01 (0.97–1.06)	0.621
TBIL	1.00 (0.99~1.01)	0.256	1.00 (0.99–1.01)	0.813
Surgical approach (PD vs. HCAE)	0.69 (0.39–1.21)	0.197	0.87 (0.36–2.13)	0.761

**Table 4:** Multivariate logistic regression analysis of PNI grouped by quartiles.

PNI Group	PNI Range	N	Severe Complications [*n* (%)]	OR (95%CI)	*p*
Q1	≤40.9	79	38 (48.1)	1.00	
Q2	41.0~44.7	75	18 (24.0)	0.42 (0.17–0.99)	0.049
Q3	44.8~48.5	79	6 (7.6)	0.14 (0.05–0.42)	<0.001
Q4	≥48.6	75	3 (4.0)	0.03 (0.01–0.15)	<0.001
*p* for trend				-	<0.001

## Data Availability

Data for the current study are available from the corresponding author upon reasonable request.

## Supplementary Materials

The following supporting information can be downloaded at: https://www.mdpi.com/article/10.3390/curroncol33090560/s1, Table S1: 10-point Modified Surgical APGAR Score; Table S2: Procedure-specific sensitivity analysis of the PNI–mSAS model.
