# Supplementary material for: Predictive Value of Preoperative Prognostic Nutritional Index and Modified Surgical Apgar Score for Severe Postoperative Complications in Malignant Obstructive Jaundice
Source: Curr Oncol. 2026 Sep 15;33(9):560. doi: 10.3390/curroncol33090560 (PMC13605575; doi:10.3390/curroncol33090560)
Supplement: Supplementary file 1 [file curroncol-33-00560-s001.zip › curroncol-4535399-supplementary.pdf]

Supplementary Tables

Supplementary Table S1. 10-point Modified Surgical APGAR Score

Component	0 points	1 point	2 points	3 points	4 points
Lowest heart rate (/min)	>85	76–85	66–75	56–65	≤55
Lowest mean arterial pressure (mmHg)	<40	40–54	55–69	≥70	—
Estimated blood loss (mL)	>1000 or transfusion	601–1000	101–600	≤100	—

The total mSAS ranges from 0 to 10 points. A higher score indicates a more favorable intraoperative physiological profile.

Supplementary Table S2. Procedure-specific sensitivity analysis of the PNI–mSAS model

Procedure	PNI OR (95% CI)	PNI P	mSAS OR (95% CI)	mSAS P	AUC (95% CI)
PD	0.75 (0.64–0.88)	<0.001	0.09 (0.04–0.21)	<0.001	0.959 (0.921–0.997)
HCAE	0.74 (0.63–0.87)	<0.001	0.54 (0.37–0.79)	0.001	0.901 (0.837–0.965)

Abbreviations: PNI, prognostic nutritional index; mSAS, modified Surgical Apgar Score; PD, pancreaticoduodenectomy; HCAE, hepatectomy with extrahepatic bile duct resection; AUC, area under the receiver operating characteristic curve; CI, confidence interval.
